# Rhabdastrellic Acid-A Induced Autophagy-Associated Cell Death through Blocking Akt Pathway in Human Cancer Cells

**DOI:** 10.1371/journal.pone.0012176

**Published:** 2010-08-17

**Authors:** Dan-Dan Li, Jing-Feng Guo, Jia-Jia Huang, Lin-Lin Wang, Rong Deng, Jian-Nan Liu, Gong-Kan Feng, Ding-Jun Xiao, Song-Zhi Deng, Xiao-Shi Zhang, Xiao-Feng Zhu

**Affiliations:** 1 State Key Laboratory of Oncology in South China, Cancer Center, Sun Yat-Sen University, Guangzhou, China; 2 The Affiliated He Xian Memorial Hospital of Southern Medical University, Guangzhou, China; 3 Guangzhou Institute of Chemistry, Chinese Academy of Sciences, Guangzhou, China; INMI, Italy

## Abstract

**Background:**

Autophagy is an evolutionarily conserved protein degradation pathway. A defect in autophagy may contribute to tumorigenesis. Autophagy inducers could have a potential function in tumor prevention and treatment.

**Methodology/Principal Findings:**

Our results showed that Rhabdastrellic acid-A, an isomalabaricane triterpenoid isolated from the sponge Rhabdastrella globostellata, inhibited proliferation of human cancer cell lines Hep3B and A549 and induced caspase-independent cell death in both the cell lines. Further investigation showed that Rhabdastrellic acid-A induced autophagy of cancer cells determined by YFP-LC3 punctation and increased LC3-II. The pretreatment with autophagy inhibitor 3-MA inhibited Rhabdastrellic acid-A-induced cell death. Knockdown of autophagy-related gene Atg5 inhibited Rhabdastrellic acid-A-induced cell death in A549 cells. Also, phospho-Akt and its downstream targets significantly decreased after treatment with Rhabdastrellic acid-A in both cancer cell lines. Transfection of constitutive active Akt plasmid abrogated autophagy and cell death induced by Rhabdastrellic acid-A.

**Conclusions/Significance:**

These results suggest that Rhabdastrellic acid-A could induce autophagy-associated cell death through blocking Akt pathway in cancer cells. It also provides the evidence that Rhabdastrellic acid-A deserves further investigation as a potential anticancer or cancer preventive agent.

## Introduction

**Figure 1:**
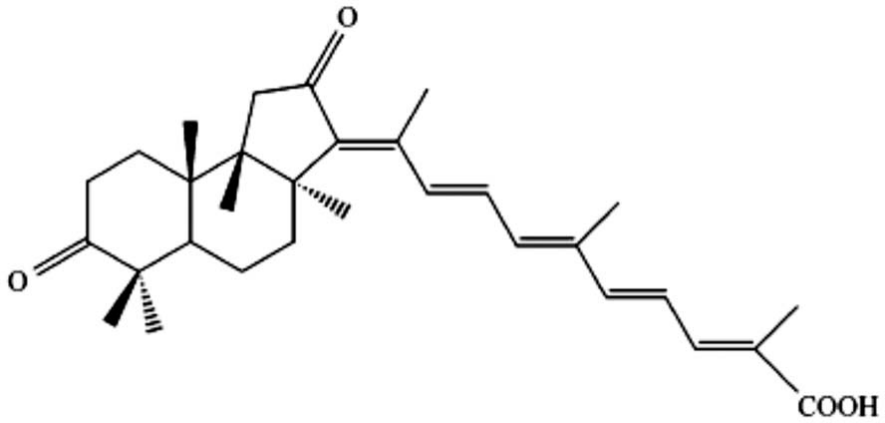
Molecular structure of Rhabdastrellic acid-A.

Marine sponges have been proven to be a particularly fruitful source of unusual terpenoids[1], [2]. Rhabdastrellic acid-A (Fig. 1), an isomalabaricane triterpenoid, was isolated from the yellow sponge Rhabdastrella globostellata (Carter) collected from the South China Sea near Hainan Island, People's Republic of China. Its structure was established on the basis of UV, IR, MS, ^1^H-NMR, ^13^C-NMR, and 2D NMR spectrometry [3], [4]. The relative and absolute stereochemistries were solved by NOESY and CD studies, respectively. It has been reported that Rhabdastrellic acid-A can inhibit growth of cancer cell line HCT-116. Tasdemir et al. found that stellettin B and E, two terpenoids from Rhabdastrella globostellata with structure similar to Rhabdastrellic acid-A, preferentially inhibit p21−/− HCT-116 cell growth [5]. Other isomalabaricane triterpenoids were found to induce reactive oxygen species (ROS), decrease mitochondrial membrane potential, increase the levels of Bax and cytochrome c, decrease the levels of Bcl-2 and mediate a caspases-3 apoptotic pathway, but the molecular mechanisms responsible for triterpenoids-induced cell death have not been elucidated yet[5]. Also, our investigation showed that Rhabdastrellic acid-A induced apoptosis in HL-60 cells [4], but the development of apoptotic features in Hep3B and A549 cells following exposure to Rhabdastrellic acid-A was not observed.

Autophagy is a lysosomal degradation pathway that is essential for survival, development, and homeostasis[6]. Autophagy principally serves an adaptive role to protect organisms against diverse pathologies, including infections, cancer[7], neurodegeneration[8], aging, and heart disease. Autophagic cell death has morphologic and biochemical features distinguishing it from both apoptosis and necrosis. At the early stage of tumour development, autophagy functions as a tumor suppressor^39^. Defects in autophagy lead to genomic instability and tumorigenesis. However, the tumor cells that are located in the central area of the tumor mass undergo autophagy to survive under low-oxygen and low-nutrient conditions. It was reported that autophagy protected some cancer cells against anticancer treatments by blocking the apoptotic pathway. By contrast, other cancer cells could undergo autophagic cell death following cancer therapies [9].

The role of autophagy in mediating the cellular response to stress has been examined by interferencing the expression of mammalian orthologues of the ATG gene [10]. For example, Beclin 1 (BECN1; also known as Atg6) is one component of a complex including the class III phosphotidylinositol-3-kinase that is stimulatory for autophagy. Beclin 1 (ATG6) is also known to interact with Bcl-2 family members, Bcl-2 and Bcl-xL[11], [12]. The induction of Beclin 1 expression can be found during autophagy in various cell types[13]; and ATG5 is required for autophagy but can also induce cell death via its interaction with Fas associated protein through death domain[14]; Therefore, the results of these important experiments may not at the moment be taken as a final evidence for the role of autophagy as either a perpetrator or a damage-ameliorating agent during stress, but more as a demonstration of the important roles its regulators are playing in cellular homeostasis and tumorigenesis[15].

A number of signaling pathways are involved in regulation of autophagy. One of the central regulators of autophagy is the target of rapamycin, TOR kinase, which is the major inhibitory signal that suppresses autophagy in the presence of growth factors and abundant nutrients. The class I PI3K/Akt signaling molecules link receptor tyrosine kinases to TOR activation and thereby repress autophagy in response to insulin-like and other growth factor signals[16]. Some of the other regulatory molecules that control autophagy include 5′-AMP-activated protein kinase (AMPK), which responds to low energy; the eukaryotic initiation factor 2α (eIF2α), which responds to nutrient starvation, double-stranded RNA, and endoplasmic reticulum (ER) stress[17], [18]. The suppression of autophagic cell death by caspase-8 in mammalian cells indicated that caspases can regulate both apoptotic and non-apoptotic cell death[19].

For the past several decades, researchers have focused on the central role of Akt in many human cancers[20], [21], [22], [23], [24], [25]. Akt signaling pathway has emerged as a central route for regulating multiple cellular processes, including survival and proliferation of many cell types. To date, many human cancers show hyperactivation of Akt kinases, thereby inhibition of Akt pathway is considered as a promising strategy for cancer treatment[26], [27], [28], [29], [30], [31], [32].

In the present study, we explored that the possibility that Rhabdastrellic acid-A killed human cancer cells via the induction of autophagy. We found that Hep3B and A549 cells treated with Rhabdastrellic acid-A underwent morphologic and biochemical changes consistent with the induction of autophagy and cell death. The mechanism of autophagy induction by Rhabdastrellic acid-A is associated with inhibition of Akt pathway.

## Results

### 1. Rhabdastrellic acid-A induced caspase-independent cell death in Hep3B and A549 cells

**Figure 2:**
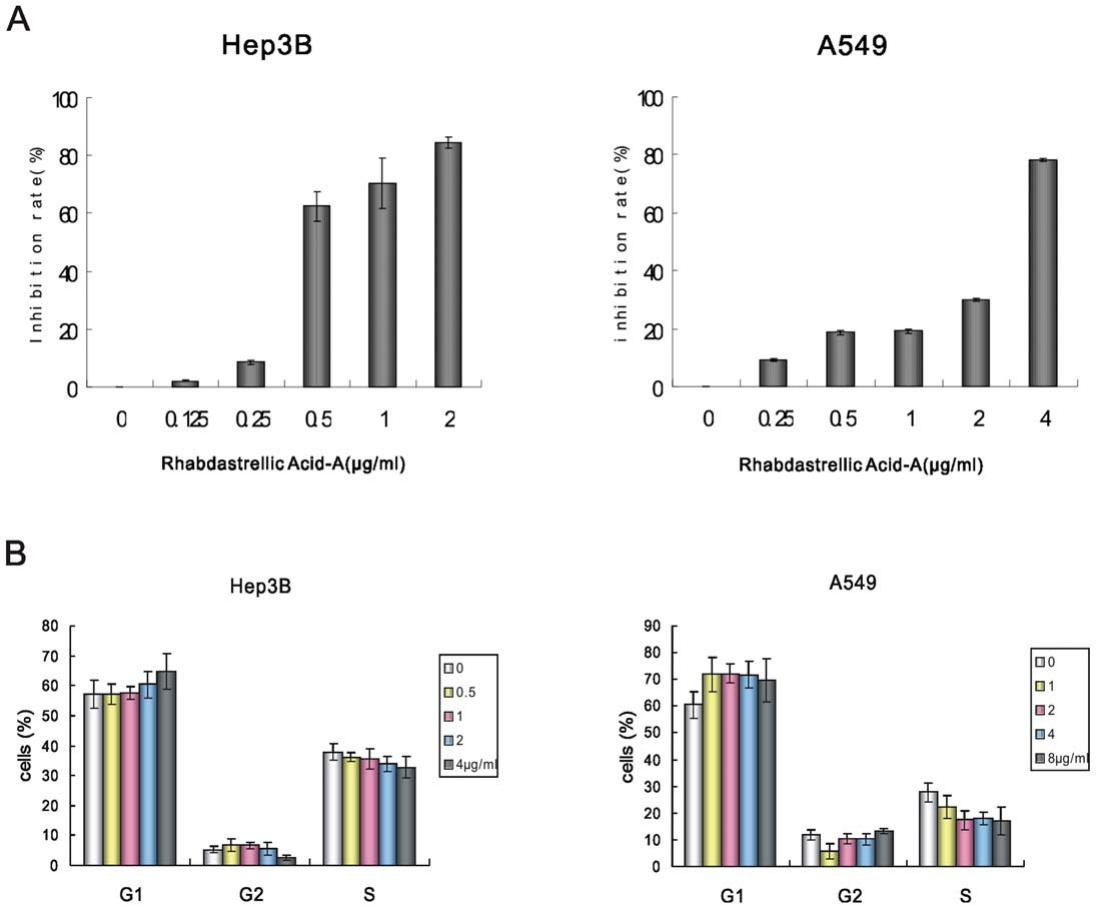
Growth inhibition of Rhabdastrellic acid-A on Hep3B and A549 cells. A. Cancer cells were plated at the density of 8000 cells/well in a 96-well plate. The stock of Rhabdastrellic acid-A was added to wells. Cells were cultured for 3 days. MTT assay was conducted. B. Hep3B and A549 cells were treated with indicated concentrations of Rhabdastrellic acid-A for 48 h and analyzed for the DNA content by flow cytometry. The results were representative of 3 experiments.

The treatment of Hep3B and A549 cells for 3 d with various concentrations of Rhabdastrellic acid-A resulted in inhibition of cell growth in a dose-dependent manner. IC_50_ value was 0.42 µg/mL and 2.50 µg/mL (Fig. 2A). Inhibition of cell growth could be the results of the induction of apoptosis, autophagy and/or cell cycle arrest. Hep3B and A549 cells treated with various concentrations of Rhabdastrellic acid-A for 48 h and analyzed for the cell cycle by flow cytometry. Fig. 2B showed that Rhabdastrellic acid-A did not induce cell cycle arrest in Hep3B and A549 cells within these concentration ranges at 48 h. Thereby, we investigated whether Rhabdastrellic acid-A could induce apoptosis or autophagy-associated cell death in Hep3B and A549 cells.

**Figure 3:**
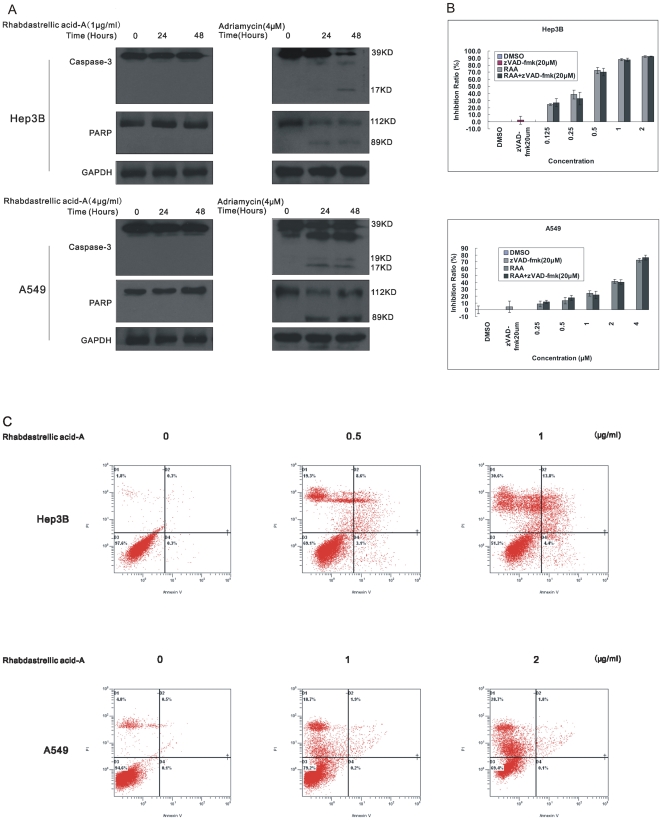
Rhabdastrellic acid-A induced caspase-independent cell death in Hep3B and A549 cells. A. in Hep3B and A549 cells, Cell lysates were prepared from 0.1% DMSO, 1 µg/mL Rhabdastrellic acid-A or 4 µM adriamycin for 24 h and 48 h. Western blot analysis was conducted with caspase-3 and PARP antibodies. B. Cells were incubated with various concentrations of Rhabdastrellic acid-A in the presence or absence of 20 µM z-VAD-fmk for 3 d. MTT assay was conducted. Results (mean±S.E.) represent the average of three experiments. C. Hep3B and A549 cells were treated with 0 to 4 µg/mL Rhabdastrellic acid-A for 48 h. After treatment, the cells were collected sequentially and stained with Annexin V and PI and analysed by the flow cytometry assay.

Many kinds of anticancer agents function to induce apoptosis of cancer cells which is characterized by caspase-3 and PARP cleavage. To determine whether the cell death was caspase-dependent, we first detected the cleavage of caspase-3. Using immunoblotting analysis, the cleavage of caspase-3 in both cancer cells was not observed following treatment with Rhabdastrellic acid-A. The cleavage of PARP was also undetectable (Fig. 2A). The cleavage of caspase-3 and PARP was also detected in both cell lines treated with adriamycin. The results showed that pro-caspase-3 was cleaved to yield a 17 KDa fragmentation and PARP was cleaved into 89 KDa fragmentation following adriamycin treatment (Fig. 3A). Furthermore, the pan-caspase inhibitor, z-VAD-fmk, did not inhibit Rhabdastrellic acid-A induced cell death (Fig. 3B). To further confirm that Rhabdastrellic acid-A mainly induced non-apoptotic cell death, Hep3B and A549 cells exposed to various concentrations of Rhabdastrellic acid-A were analyzed at 48 h intervals by Annexin V-FITC/PI staining assay. As shown in Fig. 3C, the highest apoptotic rates were 4.4% in Hep3B cells and 0.2% in A549 cells after treatment. These results strongly indicated that Rhabdastrellic acid-A induced a caspase-independent cell death in Hep3B and A549 cells.

### 2. Autophagy-associated cell death induced by Rhabdastrellic acid-A in cancer cells

#### Rhabdastrellic acid-A induces the formation of autophagosomes

**Figure 4:**
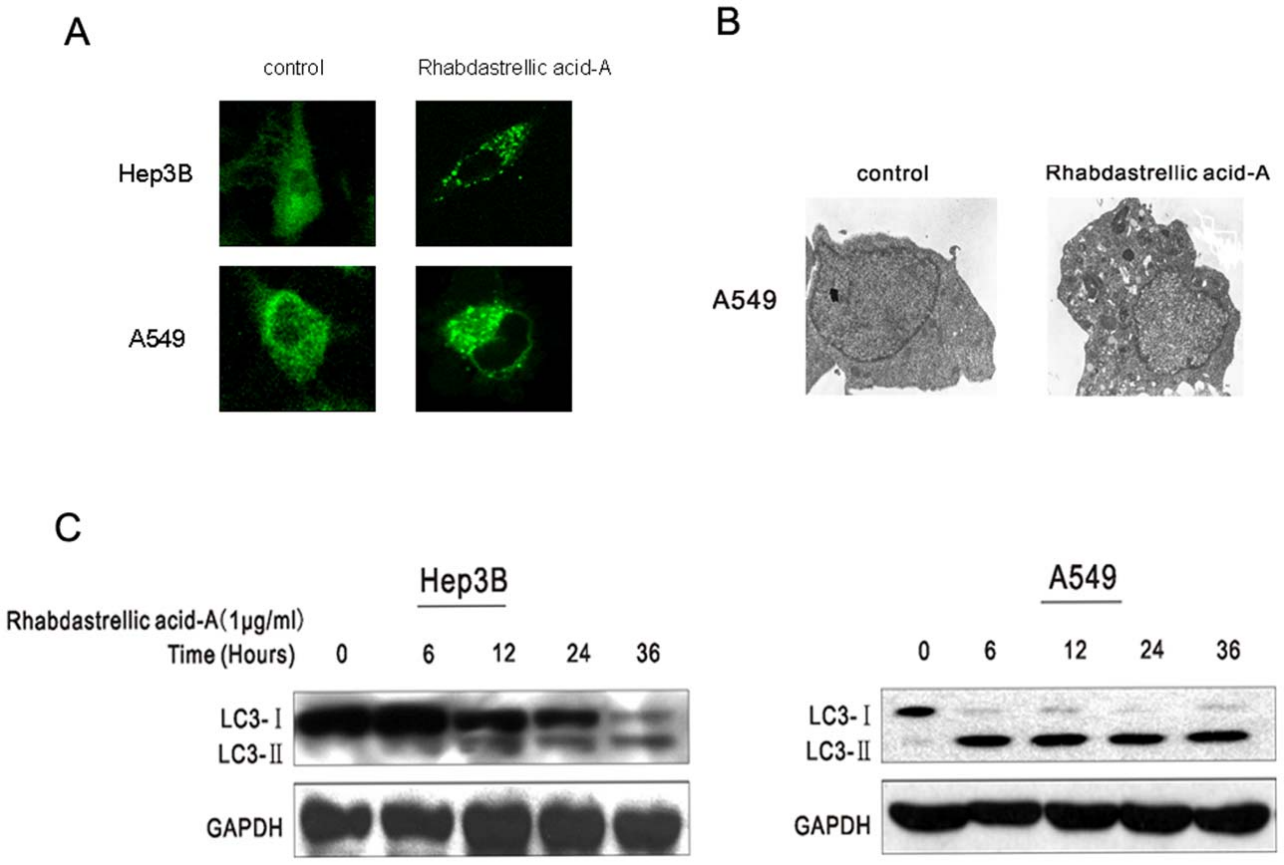
Rhabdastrellic acid-A induced autophagy in Hep3B and A549 cells. A. Hep3B and A549 cells were transfected with an expression construct for LC3 fused to yellow fluorescent protein (YFP-LC3) for 24 h. Thereafter, cells were treated with or without 1 µg/mL or 4 µg/mL Rhabdastrellic acid-A for 36 h, and visualized under a confocal microscope. B. Electron micrograph showing autophagic vacuole of A549 cells following 4 µg/mL Rhabdastrellic acid-A treatment. C. Rhabdastrellic acid-A time-dependently induced the formation of LC3-II, a marker for autophagy. Hep3B and A549 cells were treated with 1 µg/mL Rhabdastrellic acid-A for the indicated times. Lysates were analyzed by immunoblotting with LC3 antibody.

The membrane-associated light chain 3 protein, MAP LC3 (Atg8), localizes to the elongation membrane and stays in the membrane until autophagosome maturation. It is a key marker for autophagy. Upon induction of autophagy, LC3-I is converted into LC3-II, which is most likely conjugated to phosphatidylethanolamine (PE) and tightly bound to the autophagosomal membranes forming ring-shaped structures in the cytoplasm. To measure autophagy, Hep3B and A549 cells were transfected with an expression construct for LC3 fused to yellow fluorescent protein (YFP-LC3). In DMSO-treated control cells YFP-LC3 was evenly distributed in the entire cytoplasm. After Rhabdastrellic acid-A treatment, ring-shaped structures were detectable in the cytosol, indicating the association of YFP-LC3 with autophagosomal membranes following an induction of autophagy (Fig 4A). Ultrastructural analysis of Rhabdastrellic acid-A-treated A549 cells by electron microscopy showed large autophagic vacuoles in contrast to control cells where no such vacuoles could be detected (Fig. 4B).

#### LC3-II expression induced by Rhabdastrellic acid-A in cancer cells

The amount of PE-conjugated form of LC3 (LC3-II) correlates well with the number of autophagosomes. After Rhabdastrellic acid-A treatment for the indicated times, LC3 was detected by immunoblotting analysis. The results indicated that the expression of LC3-II was gradually upregulated (Fig 4C). This confirmed the results of YFP-LC3 transfection and indicated that Rhabdastrellic acid-A could induce autophagy in Hep3B and A549 cells.

#### Autophagy-associated cell death induced by Rhabdastrellic acid-A in cancer cells

**Figure 5:**
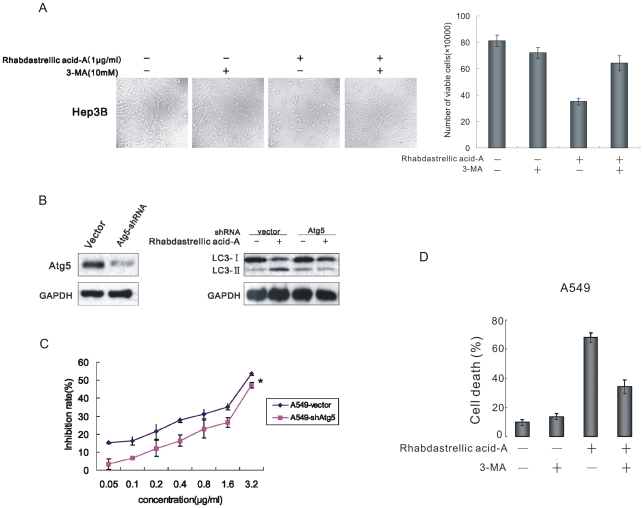
Requirement of autophagy induction for Rhabdastrellic acid-A-induced cell death. A. Hep3B cells were treated with 1 µg/mL Rhabdastrellic acid-A and/or 10 mM 3-MA for 36 h, and then examined by inverted microscope. *, p<0.05 vs. Rhabdastrellic acid-A−/3-MA-. **, p<0.05 vs. Rhabdastrellic acid-A+/3-MA-. B. Lysates from A549-Vector and A549-shAtg5 cells were analyzed by immunoblotting with Atg5 and LC3 antibodies. C. A549 vector cells and A549-shAtg5 cells were cultured at 6000 cells per well in a 96-well plate, exposed to different concentrations of Rhabdastrellic acid-A from 0.05 to 3.2 µg/mL for 72 h. The growth inhibition was detected using MTT assay. Reported values are mean ± SD of triplicate samples from a representative experiment. *P-vlaue<0.05 as compared with cells treated in the same way, but transfected without shRNA. D. A549 Cells were incubated with 4 µg/mL Rhabdastrellic acid-A and/or 10 mM 3-MA for 24 h. Cell death was quantified using flow cytometry as PI staining assay. The experiment was repeated 3 times.

To confirm that Rhabdastrellic acid-A induced autophagy-associated cell death, Hep3B cells were treated with Rhabdastrellic acid-A and/or 3-methyladenine (an inhibitor of PI3K-C3 commonly used for specific inhibition of autophagy). In contrast to control cells, after Rhabdastrellic acid-A treatment, most of the Hep3B cells appeared round, some were detached from the surface of wells and the number of cells was reduced (Fig 5A). However, the effects by Rhabdastrellic acid-A treatment were reversed by 3-MA (Fig 5A). It was suggested that the induction of cell death by Rhabdastrellic acid-A treatment was blocked when the cells were in parallel treated with autophagy inhibitor 3-MA.

shRNA-based depletion of an essential autophagy protein Atg5, effectively blocked Rhabdastrellic acid-A-induced LC3-II accumulation (Fig 5B). And the inhibition of autophagy by depletion of Atg5 inhibited Rhabdastrellic acid-A-induced cell death in A549 cells within the concentration ranges from 0.05 to 3.2 µg/mL (Fig 5C).

A549 cells exposed to 4 µM Rhabdastrellic acid-A and/or 3-MA were analyzed for cell death at 24 h by flow cytometry. Propidium iodide (PI) positive were counted as ‘dead’ cells. The cell death induced by Rhabdastrellic acid-A was suppressed when the cells were treated in combination with 3-MA (Fig. 5D).

### 3. Rhabdastrellic acid-A inhibited Akt pathway in Hep3B and A549 cells

**Figure 6:**
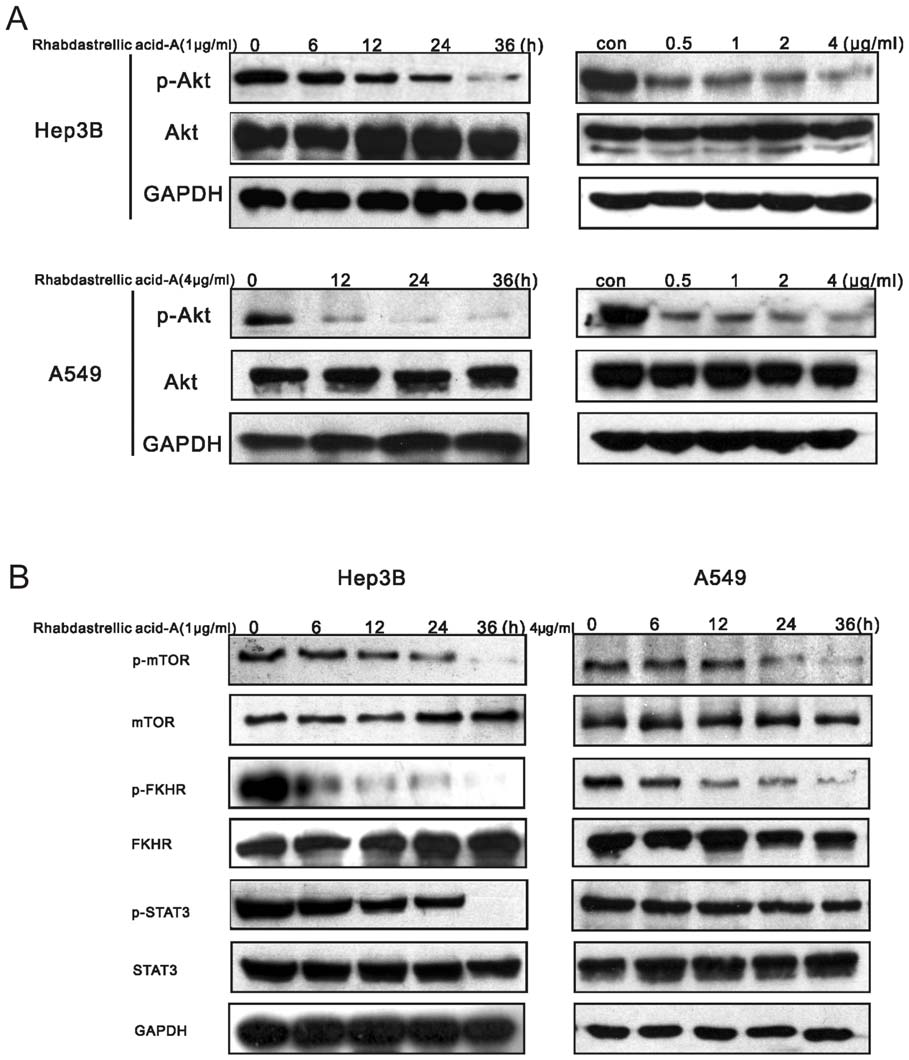
The effect of Rhabdastrellic acid-A on Akt pathway in Hep3B and A549 cells. Hep3B and A549 cells were treated with Rhabdastrellic acid-A in the indicated concentrations for 36 h or treated with 1 µg/mL or 4 µg/mL Rhabdastrellic acid-A for the indicated times. A. Cells were sequentially harvested and lysed. cell lysates were analyzed by immunoblotting with phospho-Akt or Akt antibodies. B. mTOR, phospho-mTOR, FKHR, phospho-FKHR, STAT3 and phospho-STAT3 were analyzed by immunoblotting. The experiment was performed 3 times.

Rhabdastrellic acid-A could inhibit Akt pathway in HL-60 cells[4]. Therefore, it was interesting to test whether it was the same in other cancer cells. As shown in Fig 6A, After Rhabdastrellic acid-A treatment for the indicated times or various concentrations, Rhabdastrellic acid-A inhibited the phosphorylation of Akt in Hep3B and A549 cells.

mTOR, FKHR and STAT3 are three important downstream targets of Akt pathway and play key roles in autophagy and apoptosis[33]. As shown in the results (Fig 6B), the phosphorylation of mTOR, FKHR and STAT3 had dramatic decrease after Rhabdastrellic acid-A treatment in a time-dependent manner in both cell lines. These results indicated that AKT pathway was inhibited by Rhabdastrellic aicd-A in Hep3B and A549 cells.

### 4. The induction of autophagy by Rhabdastrellic acid-A was abrogated by constitutive active Akt ectopic expression

**Figure 7:**
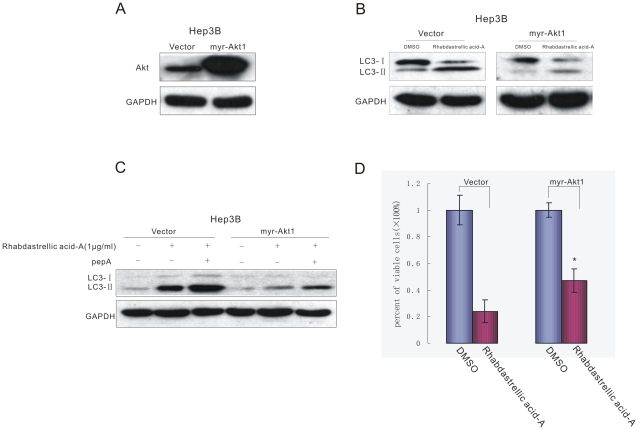
Constitutive active Akt expression could inhibit Rhabdastrellic acid-A-mediated autophagy. A. A. the positive clone stably expressed myr-Akt1 was analyzed by immunoblotting with Akt antibody. B. Hep3B cells were treated with 1 µg/mL Rhabdastrellic acid-A for 36 h in the absence or presence of constitutive active Akt ectopic expression. Then LC3 protein expression was analyzed. C. Cancer cells were treated with 1 µg/mL Rhabdastrellic acid-A and/or 10 µg/mL pepstatin A (pepA) for 36 h, Then LC3 protein expression was analyzed. D. After treatment, viable cells of two cell lines were measured. *, p<0.05 vs. Rhabdastrellic acid-A (vector).

Rhabdastrellic acid-A could inhibit Akt pathway and induce autophagy in Hep3B cells. In order to further confirm an intrinsic role of Akt in autophagy induced by Rhabdastrellic acid-A, we transfected the Hep3B cells with myr-Akt1 plasmids (activated) and selected the positive clone using G418. Compared with the control cells (Hep3B cells transfected with the vector plasmid), the exogenous Akt expression significantly increased in Hep3B/myr-Akt1 cells (Fig. 7A). Furthermore, the marker of autophagy was determined while constitutive active Akt expression in Hep3B cells. Compared with the control cells, massive induction of autophagy was observed in Hep3B cells, indicated by increase of LC3-II. However, expressing constitutive active Akt blocked increase of LC3-II (Fig 7B). Consistent with the notion that some population of LC3-II is degraded in lysosomes [34], treatment with lysosomal enzyme inhibitors Pepstatin A resulted in the accumulation of LC3-II in cancer cells (Fig 7C), this phenomenon was also impaired while constitutive active Akt ectopic expression. Furthermore, constitutive active Akt expression could rescue Hep3B cells from Rhabdasterllic acid-A-mediated growth inhibition under treatment of Rhabdastrellic acid-A (Fig. 7D). These results shown in Fig 7 indicated that constitutive active Akt expression could inhibit Rhabdastrellic acid-A-mediated autophagy.

To investigate whether other pathways are involved in autophagy induced by Rhabdastrellic acid-A, we analysed the levels of Bip, CHOP, phospho-EIF2α and phospho-AMPK in Hep3B cells following Rhabdastrellic acid-A treatment. The results showed that phosphorylation of AMPK, EIF2a and CHOP increased after Rhabdastrellic acid-A treatment in a dose-dependent manner in Hep3B cells. However, an increase of Bip was not detected (shown in Fig. S1). These results indicated that multiple pathways might be involved in Rhabdastrellic acid-A-induced autophagy.

## Discussion

The results described here demonstrate that Rhabdastrellic acid-A could induce autophagy in the human cancer cell lines. Rhabdastrellic acid-A could block Akt pathway in Hep3B and A549 cells, and constitutive active Akt expression could inhibit Rhabdastrellic acid-A-mediated autophagy. These results indicated that inhibition of Akt/mTOR pathway is involved in Rhabdastrellic acid-A induced autophagy and cell death.

Autophagy has been reported to be a process whereby double membrane vacuoles termed autophagosomes form around, engulf, cytosol and organelles. Though autophagy was reported to effect as a prosurvival response, in some instance, the cytotoxicity of arsenic trioxide, imatinib, ionizing radiation etc. to some cell types is mediated by the induction of autophagy. In those circumstances, autophagy constitutes a nonapoptotic pathway for programmed cell death. Many reports show that triterpenoids have potential cytotoxicity to cancer cell lines [35], [36]. Rhabdastrellic acid-A is one of the triterpenoids that induced non-apoptotic cell death in Hep3B and A549 cells. Therefore, we explored whether the induction of autophagy was required for Rhabdastrellic acid-A -mediated cell death in these two cancer cell lines. Autophagic cells enlarge without permeabilization of the plasma membrane, convert LC3-I to LC-II, show punctate cytoplasmic LC3 translocation and develop autophagosome. In this study, Hep3B and A549 cells exhibited morphologic and biochemical features that are characteristic of cells undergoing autophagy, not apoptosis following Rhadastrellic acid-A treatment. Also, growth inhibition of Rhabdasterllic acid-A in A549 cells was weaker than that in Hep3B cells. But Rhabdasterllic acid-A increased more LC3-II expression. Conversion of LC3-I to LC3-II can be observed in autophagy initiation. But partial LC3-II can be degraded when autophagy maturation. Only LC3-II expression could not indicate the sensitivity to autophagy.

Previous studies on mechanism underlying the regulation of autophagy in cancer cells showed that autophagy was regulated by multiple signaling pathways as diverse as the class III PI 3-kinase, and the protein kinases mTOR, ERK, and p38. Aberrant activation of Akt/mTOR signaling pathway was implicated in a variety of human malignances [20], [21], [22], [23], [24], [25], [37]. Phosphorylated Akt plays central roles in some fundamental processes, including cell proliferation, cell death, cell motility/adhesion, cell transformation, and neovascularization. When activated, Akt acts as a survival factor by preventing release of cytochrome c from mitochondria and inactivating FKHR which is known to induce expression of pro-apoptotic factors such as Fas ligand [38]. Inhibition of mTOR induces apoptosis in some types of tumor cells, whereas they trigger autophagy in other settings. Rapamycin, an inhibitor of mTOR, was reported to induce the classic autophagic cell death in cancer cells. Our previous study also showed that Rhabdastrellic acid-A inhibited PI3K/Akt pathway and induced apoptosis in human leukemia HL-60 cells[4]. So inhibition of Akt may activate some autophagy-related proteins and induce autophagy. In keeping with this finding, we observed that Rhabdastrellic acid-A could inhibit the phosphorylation of Akt in Hep3B and A549 cells. The phosphorylation of mTOR, FKHR and STAT3 also dramatically decreased after Rhabdastrellic acid-A treatment. Furthermore, ectopic constitutive active Akt expression could block autophagy induced by Rhadastrellic acid-A. Taken together, Rhadastrellic acid-A induced autophagy through inhibition of Akt/mTOR pathway.

In summary, the current studies show that Rhabdastrellic acid-A kills Hep3B and A549 cells via the induction of autophagy. Inhibition of the Akt/mTOR pathway plays a key role in Rhabdastrellic acid-A-mediated autophagy. As tumor cells frequently acquire defects in autophagy compared with normal cells, pharmacological activation of autophagy through inhibition of Akt/mTOR pathway may kill cancer cells and limit tumorigenesis, especially in cancers with defects in apoptosis. Our study also provides the evidence that Rhabdastrellic acid-A deserves further investigation as a potential anticancer agent or cancer preventive agent.

## Materials and Methods

### Drugs and reagents

Rhabdastrellic acid-A was isolated from the sponge Rhabdastrella globostellata and initially dissolved in 100% Dimethylsulfoxid (DMSO) and stored at −20°C. Methylthiazolyldiphenyl-tetrazolium bromide (MTT) and 3-methyladenine (3-MA) were purchased from Sigma Co. (Sigma Aldrich, M2128). RPMI medium 1640 were purchased from invitrogen (Invitrogen, 11875).

### Cell lines and Cell culture

Human hepatocellular carcinoma cell line Hep3B[39] and human lung adenocarcinoma epithelial cell line A549[40] were cultured in RPMI-1640 supplemented with 10% heat-inactivated fetal bovine serum, penicillin (50U/mL), and streptomycin (50 µg/mL). The cells were incubated at 37°C in humidified 5% CO2.

### MTT assay

Cells were plated in 96-well plate. The stock of Rhabdastrellic acid-A was diluted, added to the wells for the desired final assay concentration. After 3 days exposure to Rhabdastrellic acid-A, 10 µL of MTT (5 mg/L) was added to each well and incubated for four more hours, then liquid in the wells was evaporated. 100 µL of DMSO was added to each well. The absorbance was detected in the microplate reader 550 model with 565 nm wave length (Bio-rad Co., 17024). Growth inhibition was calculated and IC_50_ value was determined using the Bliss Software.

### Annexin V-FITC/PI staining assay

Cells with different treatments were collected, resuspended with 10% 1640 medium, adjusted the cell suspension concentration to approximately 1×10^6^ cells/ml, and transferred 0.5 ml of cell suspension to a microfuge tube. After added 10 µl media binding reagent and 1.25 µl Annexin V-FITC, cells were incubated at room temperature for 15 min in the dark, then centrifuged and removed media. When resuspended in 0.5 ml cold 1×binding Buffer (10 mM HEPES, 150 mM NaCl, 2.5 mM CaCl2, 1 mM MgCl2, 20% BSA pH 7.4), cells were added 10 µl propidium iodide (30 µg/ml), placing samples on ice and away from light. Apoptosis was analysed by flow cytometry ((Beckman Coulter, Fullerton, CA) at the wavelength of 488 nm immediately.

### Cell cycle detection

Cells were collected, resuspended with 1 ml precooled 70% ethanol, and fixed in 4°C overnight. After removed ethanol and added 0.5 ml staining solution (50 µg/ml PI, 100 µg/ml RNaseA, 0.2% Triton-100), cells were incubated in 37°C for 30 min in the dark. Cell cycle distribution was analysed by flow cytometry ((Beckman Coulter, Fullerton, CA) at the wavelength of 488 nm immediately.

### PI staining assay

Cells were trypsinized with 0.5 ml 0.25% trypsin for 3 min, collected and resuspended with 1 ml precooled PBS. After added 0.5 ml staining solution (50 µg/ml PI, 100 µg/ml RNaseA, 0.2% Triton-100), cells were incubated in 37°C for 30 min in the dark. Cell death was detected by flow cytometry ((Beckman Coulter, Fullerton, CA).

### Confocal microscopy and indirect immunofluorescence

Cells were grown on glass coverslips and transfected with pYFP-LC3 for Hep3B and A549 cells. 36 h after transfection the cells were treated with Rhabdastrellic acid-A and analyzed after additional 36 h. Cells were fixed with 4% paraformaldehyde in PBS for 30 min at room temperature, the slides were mounted in anti-fading solution and stored at 4°C. The coverslips were viewed with an laser-scanning confocal microscope (Olympus, FV-1000).

### Electron microscopy

Cells were fixed by immersion in a mixture of 2.5% glutaraldehyde, 2.5% paraformaldehyde and 0.05% picric acid in 0.067M cacodylate buffer (pH 7.4). Postfixation was performed in 1% osmium tetroxide followed by an overnight immersion with 0.3% uranylacetate dissolved in 50 mM maleate buffer (pH 5.0). Standard procedures for dehydration and embedding in Epon were employed. Thin sections were further stained with lead citrate, and were examined in an electron microscope (Philip, CN10).

### Western blot analysis

Lysates were prepared from 4×10^5^ cells by dissolving cell pellets in 100 µL of lysis buffer (20 mM Na_2_PO_4_ (pH 7.4), 150 mM NaCl, 1% Triton X-100, 1% aprotinin, 1 mM phenymethysulfonyl fluoride, 10 mg/mL leupeptin, 100 mM NaF, and 2 mM Na_3_VO_4_). Lysates were centrifuged at 12,000 rpm for 10 min. The supernatant was collected. The protein content was determined using the Bio-Rad protein assay. SDS-PAGE sample buffer (10 mM Tris-HCl, pH 6.8, 2% SDS, 10% Glycerol, 0.2 M DTT) was added to lysates. Lysates were heated to 100°C for 5 min, and 40 µg of protein was loaded in each well of a 4–20% SDS-PAGE gel. Resolved proteins were electrophoretically transferred to nitrocellulose and incubated sequentially with primary antibody and horseradish peroxidase-conjugated goat anti-mouse IgG (Santa Cruz, sc-2005) or goat anti-rabbit-IgG (Santa Cruz, sc-2004). After washing, the bound antibody complex was detected using LumiGLO reagent (Cell Signaling Technology, #7003) and XAR film (Kodak, XBT-1) as described by the manufactures. The following primary antibodies were used: caspase-3 antibody (Santa Cruz, sc-7272), PARP antibody (Santa Cruz, sc-7150), glyceraldehyde 3-phosphate dehydrogenase antibody (Santa Cruz, sc-47724), LC3 antibody (Novus Biologicals, NB100-2220), Atg5 antibody (Cell Signaling Technology, #2630), Phospho-Akt1/2/3(ser473) antibody (Santa Cruz, sc-7985-R), Akt1 antibody (Santa Cruz, sc-1618), Phospho-mTOR(ser2448) antibody(Cell Signaling Technology, #2971), mTOR antibody (Cell Signaling Technology, #2972), Phospho-FKHR(ser256) antibody (Cell Signaling Technology, #9461), FKHR antibody (Cell Signaling Technology, #9462), Phospho-STAT3(ser727) antibody (Santa Cruz, sc-8001-R), STAT3 antibody (Cell Signaling Technology, #9132).

### Plasmids and transfection

myr-Akt1 (activated #21–151) or empty plasmid (pUSEamp(+)#21–147) were purchased from upstate Co. Hep3B cells were seeded into 6-well plate the day before transfection. Transfections of myr-Akt1 (activated) or empty plasmid (vector) were performed with Lipofectamine 2000 (Invitrogen, 11668019) according to the protocol suggested by the manufacture. After 48 h of transfection, the positive clones were selected under G418 (1000 µg/mL).

### Constructs, Retroviral Infection, and RNA interference

For stable ATG5 siRNA expression, the retroviral vector (pSUPER. puro, a gift of Professor Musheng Zeng, Cancer Center, Sun Yat-sen University, Guangzhou, China) encoding hairpin RNA sequences was constructed. A distinct short hairpin RN (shRNA) sequences against ATG5 (shATG5) were generated and cloned into the expression vector. The ATG5 sense primers were: 5′-GGC ATT ATC CAA TTG GTT TA-3′, Vesicular stomatitis virus–pseudotyped vectors were produced by transfection of the VSV-GPG producer cell line with 5 µg DNA using lipofectamine 2000 (Invitrogen) in a six-well plate. Retrovirus-containing supernatants were collected at days 5 to 7 after transfection. Then A549 cells were infected with retrovirus-containing supernatants thrice. The positive cells stably expressing shATG5 were selected under puromycine (1 µg/mL).

## Supporting Information

Figure S1 Rhadbstrellic acid-A up-regulated p-AMPK, p-EIF2α, CHOP proteins in Hep3B cells. Hep3B cells were treated with 0–2 µg/mL Rhadbstrellic acid-A for 36 h, then the cells were collected and lysed. Cell lysates were analyzed by immunoblotting with phospho-AMPK, phospho-EIF2α, Bip and CHOP antibodies.
(2.04 MB TIF)
