# Supplementary figures and images for: Rhabdastrellic Acid-A Induced Autophagy-Associated Cell Death through Blocking Akt Pathway in Human Cancer Cells
Source: PLoS One. 2010 Aug 17;5(8):e12176. doi: 10.1371/journal.pone.0012176 (PMC2923153; doi:10.1371/journal.pone.0012176)

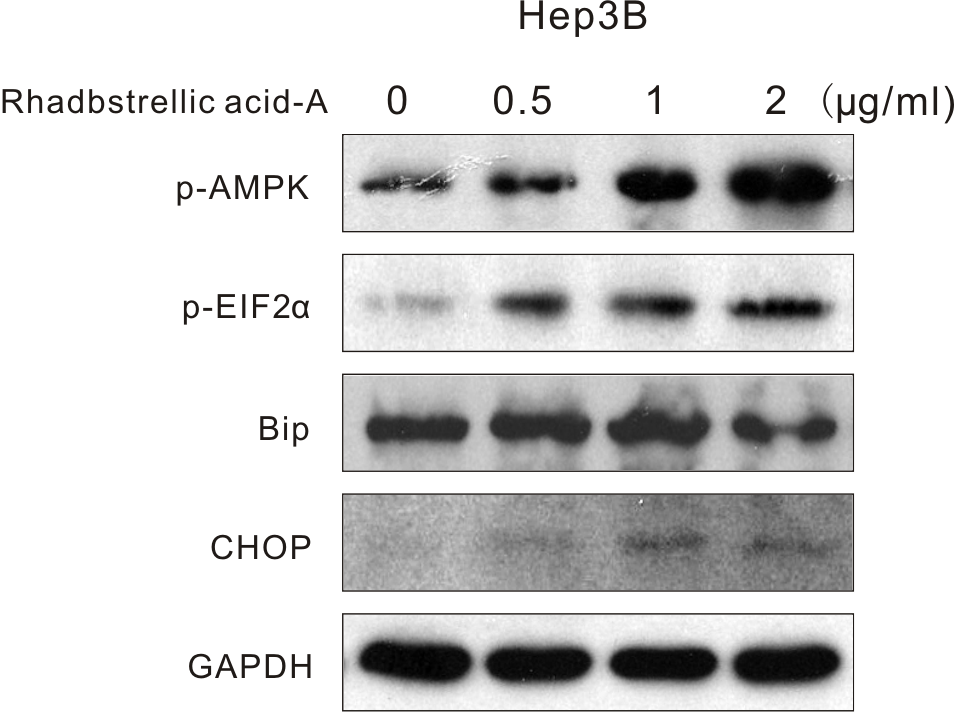

Supplement: Figure S1 — Rhadbstrellic acid-A up-regulated p-AMPK, p-EIF2α, CHOP proteins in Hep3B cells. Hep3B cells were treated with 0–2 µg/mL Rhadbstrellic acid-A for 36 h, then the cells were collected and lysed. Cell lysates were analyzed by immunoblotting with phospho-AMPK, phospho-EIF2α, Bip and CHOP antibodies. (2.04 MB TIF) [file pone.0012176.s001.tif]
